# Wireless Sensor Networks for Oceanographic Monitoring: A Systematic Review

**DOI:** 10.3390/s100706948

**Published:** 2010-07-19

**Authors:** Cristina Albaladejo, Pedro Sánchez, Andrés Iborra, Fulgencio Soto, Juan A. López, Roque Torres

**Affiliations:** DSIE, Technical University of Cartagena, Campus Muralla del Mar s/n, Cartagena, E-30202, Spain; E-Mails: cristina.albaladejo@upct.es (C.A.); andres.iborra@upct.es (A.I.); pencho.soto@upct.es (F.S.); jantonio.lopez@upct.es (J.L.); roque.torres@upct.es (R.T.)

**Keywords:** systematic review, wireless sensor networks, oceanography

## Abstract

Monitoring of the marine environment has come to be a field of scientific interest in the last ten years. The instruments used in this work have ranged from small-scale sensor networks to complex observation systems. Among small-scale networks, Wireless Sensor Networks (WSNs) are a highly attractive solution in that they are easy to deploy, operate and dismantle and are relatively inexpensive. The aim of this paper is to identify, appraise, select and synthesize all high quality research evidence relevant to the use of WSNs in oceanographic monitoring. The literature is systematically reviewed to offer an overview of the present state of this field of study and identify the principal resources that have been used to implement networks of this kind. Finally, this article details the challenges and difficulties that have to be overcome if these networks are to be successfully deployed.

## 1. Introduction

Coastal marine systems are particularly vulnerable to the effects of human activity attendant on industrial, tourist and urban development. Information and communications technologies offer new solutions for monitoring such ecosystems in real time. In response to this demand for technology, the last ten years have seen the emergence of various initiatives, from simple case studies to complex coastal observation systems designed to monitor the marine environment. These systems are composed of sensor nodes, frequently wireless, which transmit data to a sink node, in real time, on a number of physical, chemical and/or biological measurements (temperature, pH, dissolved oxygen, salinity, turbidity, phosphates, chlorophyll, *etc.*).

In this context, Wireless Sensor Networks (WSNs) [1,2] offer a new paradigm for oceanography, as in many other disciplines such as precision agriculture, environmental, engineering, *etc.* WSNs are a type of autonomous, self-organized *ad-hoc* Wireless Personal Area Networks (WPAN) composed of tens, hundreds or even thousands of smart low-rate battery-powered sensor nodes (motes). In a WSN, sensor nodes normally consist of a processor, a radio module, a power supply and one or more sensors mounted on the mote itself or connected to it. The processor controls all the node’s functions such as access to sensors, control of communications, execution of algorithms, battery saving, energy source management, *etc.*

The design, implementation and deployment of a WSN for oceanographic applications poses new challenges different to the ones that arise on land, as the impact of the marine environment on the sensor network limits and affects their development. The following are some of the most important differences:

- The marine environment is an aggressive one which requires greater levels of device protection.
- Allowance must be made for movement of nodes caused by tides, waves, vessels, *etc.*
- Energy consumption is high since it is generally necessary to cover large distances, while communications signals are attenuated due to the fact that the sea is an environment in constant motion.
- The price of the instrumentation is significantly higher than in the case of a land-sited WSN.
- There are added problems in deployment of and access to motes, the need for flotation and mooring devices, possible acts of vandalism, and others.

In spite of all these drawbacks, various case studies of monitoring of marine ecosystems using WSNs can be found in the literature [3–14]. What these solutions have in common is that they are largely designed and implemented *ad-hoc* (buoys, electronics and software), and oceanographic sensors and some other components are normally the only elements acquired from third parties.

In the scientific literature we can distinguish two broad categories of marine wireless networks depending on the data transmission medium that they use: WSNs based on radio frequency (RF) aerial communications (hereafter called Aerial-WSNs or A-WSNs) and Under-Water Acoustic Sensor Networks (UW-ASNs).

A UW-ASN [15] consists of a variable number of sensors and vehicles that are deployed to perform collaborative monitoring tasks over a given area. In underwater conditions RF does not work well because radio waves propagate only at very low frequencies (30–300 Hz) and special antennas and a bigger power supply are required. The problems that underwater acoustic networks of this kind pose include: bandwidth is severely limited; propagation delays are five orders of magnitude greater than in terrestrial radio frequency channels; higher bit error rates and temporary losses of connectivity; limited battery power because solar energy cannot be used, and so on. UW-ASNs are the best solution for viable oceanographic monitoring at great depths entailing the use of Autonomous Underwater Vehicles (AUVs) equipped with underwater sensors. Underwater networking is a relatively little-explored area although experimentation with underwater communications has been going on for several decades.

A-WSNs [1,16] consist of a set of nodes with scanty power supplies, which moreover communicate with one another by way of low-consumption radio modules. In addition, they have one or more nodes with bigger power supplies which act as sinks. These communicate with a remote station using longer-range connections (via satellite, GPRS, *etc.*). This type of network should not be confused with the ones in which each node has a large power supply and connects directly to the base station. These are isolated buoys linked to a data collection centre using satellite communications. In this case, since systems incorporate considerable computational capacities, power and communications resources they are not considered as A-WSN.

A-WSNs do not present the problems described above in the case of UW-ASNs, but they do pose other problems; for example, they have to transmit via data cables running from underwater sensors to buoys on the surface. Where these sensors are located at great depths, the problems that arise can also be serious. In short, there is no ideal solution, and the most suitable technology will depend on the particulars of each case.

The purpose of this article, following an introduction to the component elements of an A-WSN for oceanographic monitoring, is to provide an overview of the systems that have been developed in the last ten years based on the deployment of networks of this kind. The study looks basically at the most significant deployments that have been implemented world-wide. The most relevant information was gathered on each one, followed by a comparative and systematic analysis of the different solutions. As a result of that study, we have identified and summarized the main elements used (hw/sw infrastructure) when implementing the different monitoring systems, and their principal features (measurements taken, characteristics of deployment, harvesting systems, *etc.*).

Section 2 summarizes the general structure of an oceanographic A-WSN and the elements commonly used in implementing the network. Section 3 details the method used in the systematic review. Section 4 summarizes the results of the study. Section 5 presents a discussion of those results. Finally, Section 6 presents the conclusions and details the principal challenges and difficulties entailed in deploying an A-WSN for oceanographic monitoring.

## 2. Fundamentals of Aerial Wireless Sensor Networks for Oceanographic Monitoring

This section details the various components of an A-WSN network and the resources needed to deploy it for oceanographic monitoring.

### 2.1. Sensor Nodes

**Figure 1.**
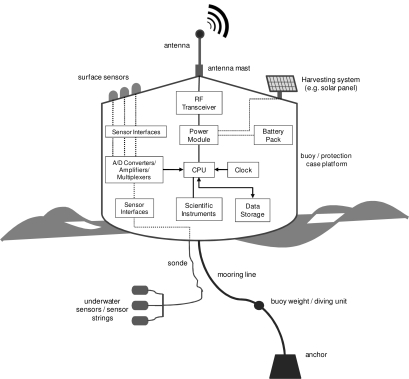
General scheme of a sensor node for oceanographic monitoring.

Figure 1 details the elements commonly used in the design and implementation of a sensor node. As we can see, it is normal to include a flotation device such as a buoy to keep part of the node out of the water. This out-of-the-water part always includes an antenna for RF transmission, optionally a harvesting system (solar panel, eolic generator, *etc.*) to supplement the power source, and in some cases one or more external sensors essentially to monitor meteorological data (windspeed, air temperature, atmospheric humidity, *etc.*). The submerged part of the node is composed of one or more sensors, which may be placed at different depths (sensor strings), a sonde to transmit the data collected to the buoy, and finally some means of anchoring the buoy to the seabed in order to prevent it from moving (due to marine currents, wind, waves, *etc.*).

The mote’s electronics include: a module for RF transmissions, a power supply regulation and management system, a set of interfaces for accessing the sensors, a module for amplification, conversion (analog to digital) and multiplexing of the data read from the sensors (surface and underwater), a FLASH-type permanent read/write memory, a clock to act as a timer, scientific instruments (e.g., improved meteorological packages, acoustic recording packages, biological samplers, *etc.*), and lastly a CPU (microprocessor) to centralize the whole process and implement the user-defined monitoring functions.

### 2.2. A-WSN General Architecture

**Figure 2.**
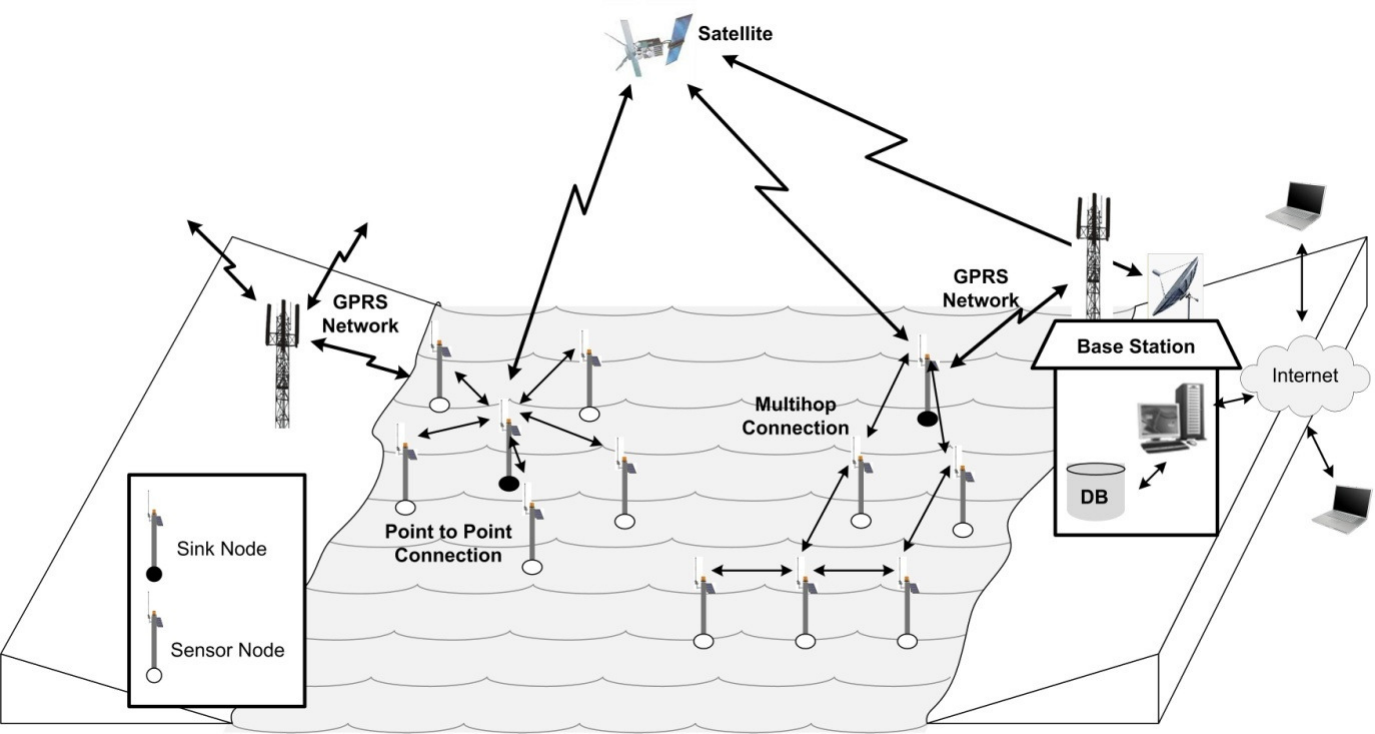
General structure of an A-WSN for oceanographic monitoring.

Figure 2 shows a general architecture of an A-WSN for oceanographic monitoring. There are two main types for inter-node communication: point-to-point *vs.* multihop. There are almost always one or more nodes that communicate directly with the base station. These act as sinks and may not have a role in the monitoring process.

The differences between deployments are essentially determined by decisions concerning:

1. the network topology;
2. the dimensions of the area to be monitored and the number of nodes used in the deployment;
3. the communication devices/protocols used and the radio frequencies chosen;
4. facilities for accessing the nodes for repair or removal (maintenance);
5. the flotation and mooring systems used;
6. the types of oceanographic sensors considered;
7. the tools for monitoring the network developed for real-time visualization of the data gathered; and
8. the electronics used for autonomous sampling of the requisite parameters and for wireless transmission to a data server.

The component elements of an A-WSN are generally common, irrespective of where they are used (wireless sensor nodes, a communications protocol, a monitoring application, *etc.*). But even so, there are major differences depending on the characteristics of the deployment. One of the most obvious such differences, as we shall see in detail later on, is the type of sensors that are to be used, which will be very specific to the environment it is proposed to monitor and the end in view.

### 2.3. Wireless Communications

Network physical topology and density are entirely application-dependant [17], so before deploying an A-WSN it is necessary to understand the environment in which it will be installed. This implies choosing the most suitable number of nodes and their absolute position inside the area to be monitored. Denser deployments improve data accuracy and provide sensor networks with more energy and better connectivity. However, at the same time a denser infrastructure can negatively affect network performance (data collisions, interferences, *etc.*) [18]. Thus, network density, physical topology and communication type all determine the logical choice of topology.

Every topology has its own characteristics, which determine whether or not it is more suitable than others in terms of attributes of network functionality such as fault tolerance, connectivity, *etc.* This means that depending on the whole set of requirements for network functionality some topologies may have to be discarded in favour of others. Of the topologies most commonly used for interconnecting nodes in a network, the logical topologies most commonly used for A-WSNs are *Tree*, *Chain*, *Partially Connected* and *Star* (indicated as point-to-point in Figure 2). It is important to note that after deployment, physical topology may change due to variations in the position, reachability (due to noise, moving obstacles, *etc.*), available energy, malfunctioning, and task details of sensor nodes [19].

For wireless communication, the sensor node incorporates a radio module, which is chosen to suit the desired range. Sometimes, in order to increase the range, range extenders for RF transceivers are incorporated, thus providing amplification to improve both output power and LNA (Low Noise Amplification). Another option, where such devices are insufficient to cover the distance, is to include a GSM/GPRS module.

For communication between sensor nodes it is possible either to develop communications protocols on the data-linking layer using different medium access mechanisms (such as TDMA, FDMA and CSMA), or else to use different wireless communication standards and technologies (Table 1).

Which technology is chosen will depend on the requirements of the A-WSN it is proposed to implement, which in turn will be determined chiefly by the amount of information that has to be sent and whether images are to be sent in real time. Another requirement that has to be considered are the maximum distances that a communications link will have to cover, as this will determine the choice of RF antenna. There are several types of antenna (omnidirectional, sector type, *etc.*) which are chosen on the basis of characteristics such as the radiation diagram, the bandwidth needed, directionality, gain, efficiency, beamwidth and the desired polarization. In the case of sensor nodes communication is more effective with omnidirectional antennas so that the radiated power is the same in all directions. This is necessary in that the movement of the sea can cause the sensor node to move rotationally, vertically or horizontally, thus altering the original position of the buoy. The drawback of this kind of antenna is that the radiated power is more dispersed and hence the range is smaller than with more directional antennas. Directional antennas need to be properly aligned and the power channelled in a single direction; this assures more range in that direction and in some cases avoids interference with other services. One important factor that must be taken into account is the height of the antenna with respect to the flotation device supporting the node, since over long distances, visual line-of-sight is not sufficient for propagation due to attenuation and so RF line-of-sight is required. The range of these nodes is affected by that height according to Fresnel Zone theory, which tells us that for true RF line-of-sight, the Fresnel Zone radius must be less than the combined height of the antennas [20].

### 2.4. Oceanographic Sensors

**Figure 3.**
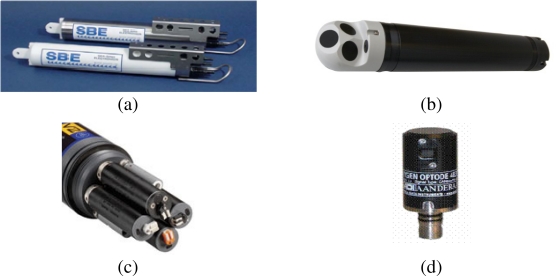
Examples of commercial sensors for oceanographic use (photographs reproduced with the owners’ permission): (a) EMS-SBE—16plus V2 SEACAT Temperature and conductivity (pressure optional) sensors; (b) NORTEK—Profiler Aquadopp (AquaPro); (c) YSI 6600V2 sonde. YSI 6136 and YSI 6025 turbidity and chlorophyll sensors; (d) AANDERAA—Oxygen Opdote 4835. Dissolved oxygen sensor.

There are many types of sensors for monitoring oceanographic parameters (physical, chemical and biological). The right choice of sensor depends on the requirements defined by the user and the requirements imposed by the characteristics of the area where they are to be deployed. These requirements include the measurement range within which the parameter is to be measured, the place where the sensor is to be deployed, sensitivity, linearity, accuracy, precision, resolution, measurement rate, power consumption and deployment time. The parameters most commonly measured in a marine environment and the measurement units used are shown in Table 2. In addition, depending on what sensor is used, it is essential to consider its position within the node and the depth at which it will be working. For example, to determine the temperature profile of a water column several sensors will have to be placed at different depths on the same vertical line. Figure 3 shows four commercial oceanographic sensors.

On the other hand, the sensor node may be equipped with surface sensors (Table 3), which are normally used to determine the state of the water surface or the atmosphere. These conditions may be important when setting up a sampling strategy. For example, in the event of bad atmospheric conditions the sensor node may decide to raise the sampling frequency to assure more precise monitoring of the environment.

### 2.5. Hw/Sw Solutions for Node Implementation

Some sensor node implementations reuse commercial solutions (MicaZ^®^, TelosB^®^, Mica2^®^, *etc.*) which come with an incorporated microprocessor, and communications electronics (radio modules, antennas, *etc.*). These motes normally come with a set of software development tools (operating system, programming languages, reusable components, *etc.*) and after-sales technical support from the supplier. When the characteristics of such commercial motes are inadequate or unsuitable, sensor nodes are commonly developed from scratch using the electronic components shown in Figure 1.

The main component is a low-power microprocessor, which is the core of the platform and is responsible for managing node operation. This microprocessor must possess certain features if it is to be suitable for use with an A-WSN: its architecture, combined with some low power modes, has to be optimized to achieve extended battery life in portable measurement applications. Also, it must include several universal serial synchronous/asynchronous communication interfaces (such as UART, I2C, SPI, *etc.*) so that the sensors can be integrated with different types of electrical signals.

The lifetime of the network depends on the autonomy of the sensor nodes. Power is normally supplied by batteries (commonly D-cell, Lithium-ion, AA or AAA batteries), which may be supplemented by harvesting systems (solar panels, eolic generators, *etc.*) to prolong the useful life of the sensor node. It is sometimes necessary to adapt the voltage between the node’s power supply and the rest of the components, by means of DC/DC converters.

Inclusion of a FLASH read/write permanent memory (SD, MMC, *etc.*) enhances the robustness of the mote by allowing data to be stored and transmitted later on when conditions permit, thus avoiding loss of information.

Another important component is a low-consumption clock operating in real time. With a clock synchronized with all the other motes, when a reading is taken from a sensor it can be stored along with the exact time of the reading. Later on, the information can be relayed to the data server, which is important when it comes to analysing the resulting data.

Sensor node software development can be done in two ways. The first and most immediate is to use specific operating systems (such as TinyOS, Contiki, MANTIS, among others) for these platforms. Although at first this way of proceeding offers advantages for the reuse of WSN libraries, it has however the drawback of limiting the hardware platforms available (e.g., due to the delay between the date in which a new microprocessor appears on the market until it is supported by operating systems). The second possibility is to develop the control of the sensor node using software libraries provided by the microprocessor manufacturer. These libraries allow users to manage both the communications and interfaces using well known standards. This option therefore offers greater possibilities for ad-hoc development since it covers more platforms and more interfaces. However, it requires much more experience on the part of developers and reuse is rather limited.

### 2.6. Monitoring Application

The information gathered by the sensor nodes has to be transmitted to a base station with a massive data storage system (relational databases are the commonest solution) which can also be used for the necessary studies using the existing oceanographic theoretical models. Having integrated monitoring tools makes it possible to maintain permanent communication with the sensor network deployed and access to the stored data via Internet. The information displayed by these tools usually consists of the number of nodes deployed, the parameters analysed, the geographical location of each node, the most recent data gathered by the sensor nodes, and a visualization of a data historical table.

**Figure 4.**
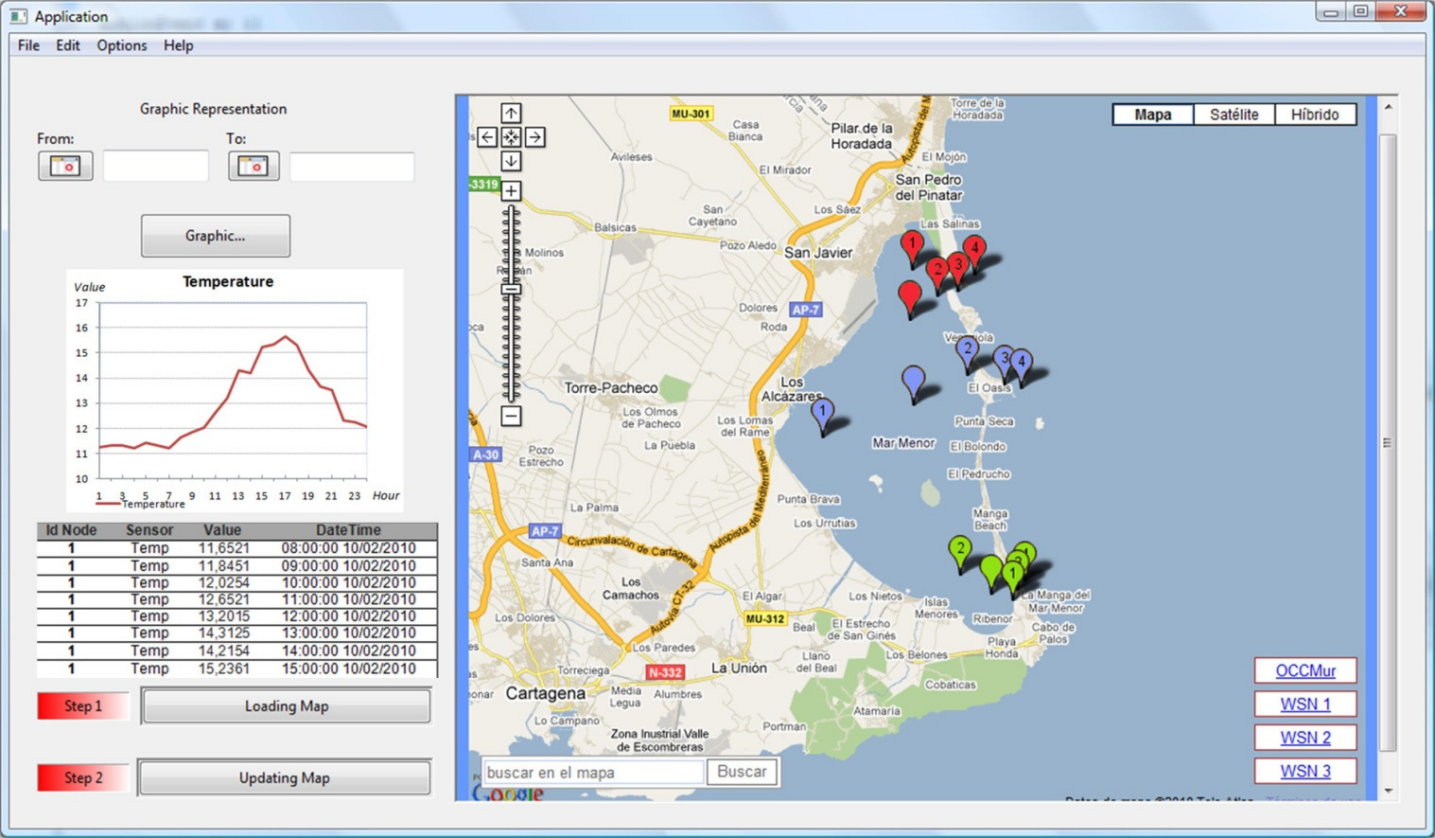
Example of an application for real-time monitoring of data from an oceanographic A-WSN.

An example of a monitoring application is shown in Figure 4, where we can see the elements that are normally incorporated: (1) a data area (left) displaying statistical data on a particular WSN subset for a user-selected time range, and (2) a two-dimensional representation of the deployment along with tools for visualizing the latest data gathered by the selected sensors in that graphic area (the example chosen uses Google Maps^®^ and the APIs necessary to include the points of interest).

## 3. Systematic Review Method

As stated in [21], “a systematic literature review is a means of identifying, evaluating and interpreting all available research relevant to a particular research question, or topic area, or phenomenon of interest”. There are several possible reasons for undertaking a systematic review of a subject of interest. The most common reasons are:

- To summarize the existing evidence relating to a piece of knowledge or a particular technology.
- To identify the gaps in an area of interest with a view to suggesting specific areas of research.
- To provide a base on which to define new lines of action on a particular subject.

In our case the main purpose of undertaking such a systematic review was to answer the following questions:

RQ1: What are the most relevant A-WSN-based oceanographic monitoring projects?
RQ2: What infrastructure is usually used in deploying A-WSNs for oceanographic monitoring?
RQ3: What is the scope of the proposals in terms of deployment, data gathered and continuity over time?

With respect to RQ1, we propose to look only at work published in the last ten years. There is very probably work published prior to A-WSNs on means of wireless monitoring of marine environments. However, we felt it reasonable to exclude these since objective RQ1 is strictly confined to A-WSN, a technology which was not available until only a few years ago. To address RQ2, we have identified and analysed the infrastructure used in the various different projects. With respect to RQ3, it is important to note that not all projects pursue the same goal. In some cases the purpose is really to find technological solutions in the field of study, while deployment serves merely to validate the results and is not a goal of the research. A systematic review must be, and seen to be, fair in order to get results and conclusions of interest and extrapolatable. In other words, a systematic review should only be undertaken on the basis of a previously-defined information search procedure or strategy. This study was carried out following the guidelines defined by [21] for the conduct of systematic reviews.

### 3.1. Search Process

The search for case studies was performed manually, analysing proceedings and journal papers since 2001. The electronic databases used were IEEE Xplore, ACM Portal, ScienceDirect, SpringerLink, CiteSeer and Google Scholar. The following three groups of search terms were used in each one:

1. “monitoring”, “environmental sensing”, “observation”
2. “WSN”, “wireless sensor networks”, “sensor”
3. “oceanography”, “marine”, “aquatic”, “coastal marine”, “oceanographic”

Each journal and conference proceedings was reviewed by three researchers (*i.e.*, Albaladejo, Iborra and Sánchez) and any papers that addressed literature surveys of any type were identified as potentially relevant. The three named researchers later agreed on what works were representative following the pre-defined inclusion/exclusion criteria.

### 3.2. Inclusion and Exclusion Criteria

Study selection criteria are intended to identify primary studies that provide direct evidence on the subject of the research. Peer-reviewed articles on the topic of interest, published between 2001 and 2010, were included. Articles were discarded if they presented one or more of the following characteristics:

- The article did not demonstrate the deployment of an A-WSN for oceanographic monitoring. To so demonstrate it had to furnish data on the marine environment to be monitored, include detailed photographs showing the design of the buoys used and the sensor nodes deployed in the area of interest. Another essential condition was that it states the physical parameters of interest for monitoring.
- The article placed more interest in the technology used, taking less interest in deployment and accordingly lacking detailed information thereon.
- The article had not been published or the congress/journal was not one of acknowledged prestige, either in the field of oceanography or in that of A-WSNs.
- Repeat articles on the same study or deployment. Where reports of a study had been published in several different journals, the most complete version of the study was included in the review.

Also, it was considered whether or not to consider as relevant case studies of oceanographic deployments in which isolated buoys linked to the data collection centre satellite were used. It was decided to exclude such systems as not really A-WSNs—that is, systems incorporating considerable computational capacities, power and communications resources and hence contradicting the essential idea of an A-WSN in which the sensor nodes are limited in power, computational capacities, and memory as stated in [1].

### 3.3. Data Collection and Analysis

The data extracted from each study were:

- The source (journal or proceedings) and full reference.
- Identification details of the monitoring project (name, duration, *etc.*).
- The organization that carried out the work (universities, research centres, *etc.*).
- Technical aspects concerning the A-WSN that was implemented (number of nodes, sensors incorporated, power supply system, microprocessor used, communications protocols, network range, topology, radio frequencies used and autonomy).
- Aspects relating to the deployment (year in which it was implemented, location that was monitored, time devoted to data collection and system testing).

Three researchers did the data extracting and the rest checked the resulting data. Albaladejo coordinated the data extraction and the checking, which involved all the authors of this article. In the event of disagreements, the data were discussed in detail until agreement was reached.

## 4. Systematic review results

This section summarizes the results of the study.

### 4.1. Search Results

Tables 4 and 5 show the results of the search procedure. Initially, twenty articles were identified with the referenced search procedure, but five of them were simplified versions of two. Moreover, three out of the twenty made no explicit mention of using A-WSNs, leaving us in doubt as to whether they had actually used such an infrastructure. Then again, one article was discarded because it was based on underwater acoustics, which falls outside the scope of this paper as noted in the introduction. Another of the articles was excluded from the survey because it did not specify whether the system had actually been implemented. In this way we identified twelve unique studies. Half of the articles were published in the proceedings of international congresses and the other half in journals of acknowledged prestige. Table 6 lists the conferences and journals identified in the survey, showing that articles of interest have been published since 2002.

### 4.2. Quality Evaluation

To assess the quality of the data reported, it was decided to e-mail the first two authors of each publication asking them to check or complete the information gathered. We received replies to seven out of twelve such requests. In some cases the reply furnished further details of the implementation. In general terms, there were no significant discrepancies between the data gathered from the publications and the information received by e-mail.

## 5. Discussion

This section discusses the replies received to the questions that were initially put.

### 5.1. What are the most Relevant A-WSN-based Oceanographic Monitoring Projects?

**Figure 5.**
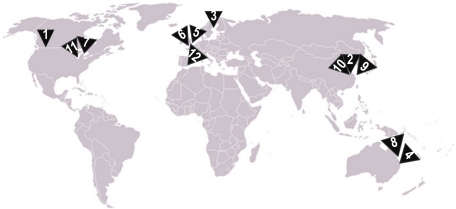
World map showing the location of the projects reviewed.

Figure 5 shows the world-wide distribution of the projects selected with this systematic review. Note that they are distributed between North America, Europe, China and Australia, reflecting the global pattern of interest in advances in this field.

Universities took part in 11 of the 12 studies and specialized marine research centres participated in half of them.

### 5.2. What is the Infrastructure usually used in Deploying A-WSNs for Oceanographic Monitoring?

All the deployments that were implemented were for monitoring of coastal waters. The commonest parameters measured were T, pH, turbidity and Dissolved Oxygen (DO). The commonest topologies were *Star* and *Partially Connected*. The most commonly used radio frequency band is 2.4 GHz ISM. And finally, eleven of the twelve systems used batteries, and six of the twelve considered using solar panels.

Access to and upkeep of the component elements of an oceanographic A-WSN entail a number of added difficulties on top of the ones that arise on land. It is therefore essential to define a system maintenance strategy to minimize the attendant costs and preserve proper functioning.

Maintenance of a network once in place essentially has to address three clearly distinct aspects:

(1) maintenance of sensorization and communication elements (antennas) to prevent loss of functionality or deterioration (biofouling, calibration, orientation, *etc.*); (2) maintenance of the power supply (batteries, solar panels, *etc.*); and (3) maintenance of the network infrastructure and topology (e.g., performance degradation, synchronization of clocks, vandalism, *etc.*). Following are details of the survey results concerning these aspects:

1. When the buoy system is used for long-term observation, the sensors are susceptible to biofouling (microbial and algal films). Special care must therefore be taken with the quality of the instruments used, since the short-term effects of biofouling can be considerable (the quality of the measurements can sometimes be compromised in less than a week). Many techniques have been studied to prevent biofouling on materials [22]. The following need to be taken in to account when considering biofouling protection for oceanographic sensors:
  - It should not affect measurement or the environment.
  - It should not consume too much energy in order to maximize the autonomy of the monitoring system.
  - It should be reliable even in aggressive conditions.
  Surface treatment based on antifouling paints is useful mainly to protect the sensor housing. UV radiation is a highly promising alternative that is non-invasive of the marine environment; however, it is not viable with the technology currently available owing to the high energy input that it requires. Another means of protection is to cover the most sensitive elements of the sensor with copper, but again the cost is high. In a word, this is a most important factor that sensor node designers need to take into account. Sensor elements normally have to be calibrated with non-portable instruments, which means that the node has to be accessed and the sensor removed for calibration in the laboratory. Maintenance operations relating to relocation of the sensor node can be performed without withdrawing the node from the network.
2. The node’s power supply will require short-, medium- or long-term maintenance depending on the elements used. In normal conditions batteries limit operability to a matter of months, or even weeks [23]. It is therefore necessary to consider the use of renewable energies such as solar, eolic, wave or tidal power to significantly reduce system maintenance requirements. The most widely used is solar power because light energy is available practically constantly, and because of the accumulated experience in integrating solar panels as a supplementary energy source in A-WSN deployments [23].
3. One of the main challenges for A-WSNs as regards network topology and infrastructure is to achieve a network that functions without sacrificing originally-defined requirements in respect of performance, sensor coverage and connectivity [18]. In oceanographic applications the possibilities of perturbations in the original physical topology are heightened by the fact that the sea is a medium in constant movement (waves, tides, *etc.*). It is therefore desirable to use techniques that make it possible to monitor the status of the network and possible changes in its original (ideal) deployment. Several solutions have been proposed in the literature addressing these issues [24]. At the same time, considerable effort has been devoted to the design of energy efficient message delivery and data retrieval methods [25]. Theoretically, point-to-point systems are the most reliable because there is only one point of failure in the topology (the host). Moreover, the system can be made more robust by adding more hosts. However, if the signal range is too short it may be necessary to consider other topologies that offer wider coverage while minimizing the risks of system breakdown through the failure of a single node [19].

**Figure 6.**
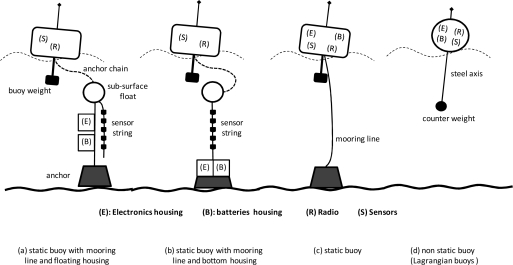
Most representative configurations of buoys used in A-WSNs.

In all the cases reviewed, the design of the flotation device (buoy) was seen as crucial. Firstly, it is important to analyse the component elements of the node and their weights, since a given weight at a considerable distance from the buoy’s line of flotation can cause instability, producing oscillations to avoid capsizing. Secondly, if such elements as batteries and electronics are close to the waterline, the space where they are contained requires more insulation. Then there is the Fresnel effect, which means that the communications antenna must be raised as high as possible to assure better robustness and quality of node communications. We identified a number of buoy implementations on the basis of the location of their components (electronics, radio, batteries, sensors) and mooring system. Figure 6 shows the most representative sensor locations identified in the survey.

### 5.3. What is the Scope of the Projects in Terms of the Deployment, the Data Collected and the Deployment Time?

Most of the A-WSNs reviewed cover relatively small marine areas only a few kilometres across (not more than 20), and the distance between nodes is in a range of 100–250 m. As to time resolution, in most of these cases the data were sampled every 5 to 10 min. This indicates a higher space-time resolution than can be achieved with the networks of buoys used in coastal oceanographic observatories. There, the spatial resolution is a matter of kilometres and the time resolution one of hours or days. On the other hand, coastal observatories normally cover a much larger area, as much as several hundred kilometres across.

Lastly, it is worth noting that practically all of the deployments were implemented for a short period of time, lasting at most three weeks, except in the case of number 11 (GLUCOS Project), in which monitoring was carried on for whole semesters. What this shows is that most of these deployments were purely experimental and that there is still a long way to go before we can have more permanent facilities.

## 6. Conclusions

The studies reviewed in this survey show that aerial wireless sensor networks (A-WSNs) are an important technological breakthrough for monitoring some oceanographic processes in which it is necessary to achieve high space/time resolutions. Also, the investment cost of deployment of an A-WSN is smaller in terms of both time and money than classic solutions like the ones used in coastal oceanographic observatories, where higher-performance devices are employed (deep-water buoys, autonomous underwater vehicles, gliders, lagrangian buoys, *etc.*).

From this survey it is fair to conclude that the solutions devised are generally ad-hoc ones as their design depends on various influencing factors. These factors include: the characteristics of the marine environment (deep water as opposed to shallows or coastal lagoons, climatic conditions, *etc.*), the time-scale of the deployment (from monitoring for a matter of weeks or months to permanent installations), the spatial scope of the deployment (large areas with low spatial resolution as opposed to smaller areas with higher resolution), and the time resolution of data collection (sampling frequencies ranging from minutes or hours to days or weeks). Because of all these different possible scenarios, designs have to be ad-hoc and have to minimize the costs of deployment and maintenance of the network. One important factor in deployment costs is the sensors that are used, which can account for up to 70% of the total initial investment. Reducing the costs of this kind of sensors is therefore a major hurdle that will have to be overcome to enable large-scale implementation of networks of this type. Other important challenges include:

– Efficient power supply systems to cover the duration of the deployment.
– Components that guarantee appropriate levels of insulation and corrosion-proofing (IP67, IP68, IP69K, *etc.*). It will be essential to obtain designs that minimize the number of connectors used, since these are especially sensitive to corrosion in a marine environment.
– Design of buoys with ready access to their components for maintenance and eventual dismantling. This essentially entails replacing power supply systems, replacing or calibration of the sensors used, and dismantling of the system once the monitoring task is concluded.
– Continued improvement of communications systems (antennas and radio modules) so that they are more reliable and guarantee communication between sensor nodes in adverse weather conditions.
– Buoy designs that minimize the impact of the networks deployed on the environments that are monitored. The presence of floating buoys can be a problem in areas with busy sea traffic. Also, buoys can be stolen or vandalized and therefore need to have means of protection (concealed GPS positioning systems, alarms, *etc.*).

The results indicate that A-WSN implementations for oceanographic monitoring are viable. Although research on the use of A-WSNs for oceanographic monitoring is still in its infancy, it points to several exciting challenges which require further interdisciplinary collaboration.

## Figures and Tables

**Table 1.** Wireless communication technologies.

**Technology**	**Standard**	**Description**	**Throughput**	**Range**	**Frequency**
WiFi	802.11a	System of wireless data transmission over computational networks.	11/54/300 Mbps	<100 m	5 GHz
	802.11b/g/n				2.4 GHz

WiMAX	IEEE 802.16	Standard for data transmission using radio waves.	<75 Mbps	<10 km	2–11 GHz
					3.5 GHz: Europe

Bluetooth	IEEE 802.15.1	Industrial specification for WPAN which enables voice and data transmission between different devices by means of a secure, globally free radio link (2.4 GHz).	v. 1.2: 1 Mbps	Class 1: 100 m	
			v. 2.0: 3 Mbps	Class 2: 15–20 m	2.4 GHz
			UWB: 53–480 Mbps	Class 3: 1 m	

GSM		Standard system for communication via mobile telephones incorporating digital technology	9.6 Kbps	Dependent on cellular network service provider	900/1800 MHz: Europe
					1900 MHz: USA

GPRS		GSM extension for unswitched (or packaged) data transmission.	56–144 Kbps	Dependent on cellular network service provider	2.5 GHz

	IEEE 802.15.4	Standard defining the physical level and control of medium access of WPANs with low data transmission rates.	20 Kbps: 868 MHz: Europe		
			40 Kbps: 915 MHz: Americas	<100 m	868/915 MHz and 2.4 GHz.
			250 Kbps: 2.4 GHz: Worldwide		

ZigBee	IEEE 802.15.4	Specification of a set of high-level wireless communication protocols for use with low-consumption digital radios, based on WPAN standard IEEE 802.15.4.	250 Kbps: 2.4 GHz: Worldwide	<75 m	2.4 GHz.

**Table 2.** Common oceanographic sensors.

**Measured Parameter**	**Unit**
*Temperature*	°C, °F
*Pressure*	mmHg
*Salinity (Conductivity)*	g/L
*Water speed*	m/s
*Turbidity*	FTU (Formazin Turbidity Unit)
	NTU (Nephelometric Turbidity Units)
	JTU (Jackson Turbidity Unit)
	mg/L SiO_2_
*Chlorophyll*	μg/L
*Dissolved oxygen*	mg/L
*Nitrate*	mg/L
*pH*	pKa
*Swell*	Height: (metres)
	Direction:(degrees)
*Blue-Green Algae Phycocyanin*	Relative Fluorescence Units
*Ammonium/ammonia*	mg/l-N
*Chloride*	mg/L
*Rhodamine*	μg/L
*Hydrocarbons*	ppm

**Table 3.** Surface sensors.

**Measured Parameter**	**Unit**
*Air temperature*	°C, °F
*Air pressure*	mb
*Wind speed*	m/s
*Wind direction*	degrees
*Precipitation*	mm, inch
*Atmospheric pressure*	mmHg
*Relative humidity*	%RH
*Solar radiation*	W/m^2^
*Surface salinity*	ppt
*Surface conductivity*	S/m

**Table 4.** Systematic review results: Organizational aspects.

**N^o^**	**Country**	**Project**	**Organization**	**Year of the deployment**	**Period of tests**	**Place of tests**	**Is the project working?**	**Ref.**
1	USA	LakeNet	University of Notre Dame	2005	10 days	St. Mary’s Lake (Indiana)	No	[3]
2	China	----	College of Information Engineering, Key Lab of Exploitation and Preservation of Coastal Bioresource, School of Biosystems Eng. and Food Science	2008	6 hours	Zhejiang Province	Yes	[4]
3	Sweden	Klimat	Swedish Institute of Comp. science; Umeǻ Marine Sciences Centre, Uppsala University	2006	20 hour test at office and at Baltic Sea	Umeǻ Marine Sciences Centre; Baltic Sea	No	[5]
4	Australia	Part of SEMAT project	University of Queensland	2007	1 week	One Mile, Moreton Bay	?	[6]
5	UK	SECOAS	University College London, British Telecommunications plc, Itelisys, Kent University, Essex University, University of East Anglia	2008	2 weeks	Scroby snads, Norforlk Coast	No	[7]
6	UK	SmartCoast	University College Cork, Dublin City University, Marine Technologies Division (Ireland)	2006	3 weeks	River Lee in Cork	?	[8]
7	USA	ReCON	Great Lakes Environmental Laboratory, Thunder Bay National Marine Sanctuary, Cooperative Institute for Limnology and Ecosystems Research	2006, 2008	?	Lakes Michigan, Huron and Erie	?	[9]
8	Australia	GBROOS (Great Barrier Reef Ocean Observing System)	Australian Institute of Marine Science, James Cook University, University of Melbourne, University of Queensland, University of Sydney, Australian Museum. Australian Institute of Marine Science (AIMS)	2008–2011	2 years (8.6 million observations collected)	Great Barrier Reef, North-East Coast	Yes	[10]
9	China	----	Hangzhou Dianzi University, Environmental Science Research & Design Institute of Zhejiang Province	2008	1 month	Artificial lake at HangZhou Dianzi University	?	[11]
10	China	OceanSense	Ocean University of China	2006		Costal waters of China	?	[12]
11	USA	GLUCOS	University of Wisconsin, Milwaukee	2007, 2008	April to June 2008	Lake Michigan off the Milwaukee coast	?	[13]
12	Spain	CMS-OOCMUR	Technical University of Cartagena	2010	1 week	Coastal Lagoon Mar Menor, Cartagena	Yes	[14]

**Table 5.** Systematic review results: Technical details.

**Nº**	**Nº of nodes**	**Hardware**	Sensors (*)	**Protocol**	**Range**	**Topology**	**Radio**	**Harvesting System**	**Autonomy**
1	8	MICA2/MDA300 modules	T, DO, and pH	Custom DARPA	All: ∼30m. Node spacing: 1 – 2 m	Star	433 MHz ISM band	2 D-cell batteries, a 12V marine battery	Two weeks
2	8	MSP430F149, I2C EEPROM	T, pH, salinity, DO and COD, Air temperature, Air humidity, Light density; 2 node inner indexes: CPU voltage, chip temperature	Zigbee	250 m	Double chain	443 MHz ISM radio	Batteries + Solar energy	∞
3	2	MSP 430F1232	T on different heights from the water surface down to the bottom	Contiki OS; GPRS	∞	Point to point	CC1100, GPRS	Battery; future: wave generator & solar cells.	Not 100%
4	10	MicaZ	Illuminance and sensor temperature	Custom adaptaive TDMA	WSN - Server: 600m. WSN: 100m. Node spacing: 10m	Star	2.4 GHz ISM band Tx power 50 mW	2 solar panels. The energy is stored in two battery packs	∞
5	10	PIC 18F452 PIC 16F76	T, pressure, turbidity, tilt, conductivity	Proprietary protocol. kOS	Node spacing: 150m. All: 2km	Multihop topology	173.25 MHz ISM band, GSM	2 alkaline D-cells	Three months
6	?	Tyndall mote ATmega128L μC	T, phosphate, DO, conductivity, pH, turbidity, water level	Zigbee, TinyOS, IEEE 1451	?	?	2.4 GHz ISM band	Batteries up to 560mAh; 3 NiMH	?
7	?	?	wind, air T, waves, water T and current profiles, chlorophyll, pH, photosynthetic active radiation, DO	IEEE802.11b	24 km	?	2.4 GHz ISM band	Solar/batteries (lead acid)	?
8	>10	Campbell Scientific Loggers	Conductivity, pressure, salinity, T, chlorophyll/fluorescence, turbidity. Meteorological station. Nortek ADCP.	TCP/IP will move to 802.11/TCP/IP	Buoys spacing: 1 km. Reef towers spacing: 2 km	?	RF411 radio (920–928 MHz), future: 802.11/WiFi	Solar/batteries	Twelve months
9	5	MSP430F1611, CC2420, CC2430 radio modules	T, pH, (future: DO, electrical conductivity rate and T)	ZigBee	?	?	2.400–2.4835 GHz	2 lithium batteries or 6 nickel-hydrogen batteries	?
10	20	TelosB	Environmental T and light intensity	?	300m x100 m	?	?	Lithium batteries	?
11	5	Single Board Computer TS-7260	T sensor string, sonde with T, conductivity, pressure, turbidity, chlorophyll A fluorescence, pH, DO	RS-485	12 km	Point to point	900MHz wireless modem	4 Lithium-Ion AA batteries	One month/an entire season
12	10	MSP430F2618, CC2520 radio module, CC2591 range extender	T, pressure and Nortek ADCP	ZigBee	All: ∼20km. Node spacing: 2 km	Star, chain, tree, mesh	2.4 GHz ISM band	6 Lithium-Ion batteries 2000mAh and 2 solar panels	Three months

(*) T: Temperature, DO: Dissolved Oxygen, COD: Chemical Oxygen Demand

**Table 6.** List of conferences and journals identified by the review.

**Publication title**	**Type (C)=Conference; (J)=Journal**	**Year**
Sensors	(J)	2002
International Workshop on Sensor and Actor Network Protocols and Applications	(C)	2004
Spatial Sciences Qld	(J)	2004
Workshop on Real-World Wireless Sensor Networks	(C)	2005
Microelectronics International	(J)	2005
Electronic Letters	(J)	2005
MTS/IEEE-OES OCEANS	(C)	2007
International conference on intelligent sensors, sensor networks and information (ISSNIP)	(C)	2007
IEEE International Workshop on Practical Issues in Building Sensor Network Applications	(C)	2007
IEEE Conf. on Local Computer Networks	(C)	2007
Environmental Engineering Science	(J)	2007
SENSEI Workshop, ICT-MobileSummit	(C)	2008
MTS/IEEE Oceans	(C)	2008
Workshop for Space, Aeronautical and Navigational Electronics	(C)	2008
ICT-Mobile Summit Conference	(C)	2008
Conf. On Embedded Networked Sensor Systems. Int. Workshop on UnderWater Networks	(C)	2009
Asian Control Conference	(C)	2009
Sensors	(J)	2009
Computers, environment and Urban Systems	(J)	2009
